# Cage Matching: Head to Head Competition Experiments of an Invasive Plant Species from Different Regions as a Means to Test for Differentiation

**DOI:** 10.1371/journal.pone.0004823

**Published:** 2009-03-13

**Authors:** Christopher J. Lortie, Michael Munshaw, Andrea Zikovitz, Jose Hierro

**Affiliations:** 1 Department of Biology, York University, Toronto, Ontario, Canada; 2 Department of Biology, York University, Toronto, Ontario, Canada; 3 Ecology and Evolutionary Biology, University of Toronto, Toronto, Ontario, Canada; 4 Conicet, University of La Pampa, Santa Rosa, La Pampa, Argentina; University of Lancaster, United Kingdom

## Abstract

Many hypotheses are prevalent in the literature predicting why some plant species can become invasive. However, in some respects, we lack a standard approach to compare the breadth of various studies and differentiate between alternative explanations. Furthermore, most of these hypotheses rely on ‘changes in density’ of an introduced species to infer invasiveness. Here, we propose a simple method to screen invasive plant species for potential differences in density effects between novel regions. Studies of plant competition using density series are a fundamental tool applied to virtually every aspect of plant population ecology to better understand evolution. Hence, we use a simple density series with substitution contrasting the performance of *Centaurea solstitialis* in monoculture (from one region) to mixtures (seeds from two regions). All else being equal, if there is no difference between the introduced species in the two novel regions compared, Argentina and California, then there should be no competitive differences between intra and inter-regional competition series. Using a replicated regression design, seeds of each species were sown in the greenhouse at 5 densities in monoculture and mixed and grown till onset of flowering. *Centaurea* seeds from California had higher germination while seedlings had significantly greater survival than Argentina. There was no evidence for density dependence in any measure for the California region but negative density dependence was detected in the germination of seeds from Argentina. The relative differences in competition also differed between regions with no evidence of differential competitive effects of seeds from Argentina in mixture versus monoculture while seeds from California expressed a relative cost in germination and relative growth rate in mixtures with Argentina. In the former instance, lack of difference does not mean ‘no ecological differences’ but does suggest that local adaptation in competitive abilities has not occurred. Importantly, this method successfully detected differences in the response of an invasive species to changes in density between novel regions which suggests that it is a useful preliminary means to explore invasiveness.

## Introduction

Understanding the success of invasive plants is not necessarily simple [1], [2]. Invasion is primarily a biogeographical issue as it involves the movement (either intentionally or accidentally) of a species from one region to another [3], [4]. The application of this filter as a means to infer differences is powerful, and there are a variety of broad applications such as (i) population-level experiments, i.e. comparison of success in home versus away regions [5], [6], assessment of variation in dominance in novel ranges [7], or gradient studies [8], [9] and (ii) individual-based tests such as evidence for differences in plasticity [10], genetics [11], [12], fitness [13], or ecotypic differentiation in morphology such as size [14], [15]. Equally fundamental to the biogeographical approach to studying invasions is the use of density following movement to a region to infer invasiveness. Relative changes in the population density of an introduced species (i.e. increases) in a novel region is arguably the primary, yet informal means, to infer that a plant species is invasive. Yet, the importance of density as a regulating process within each novel range wherein an introduced species is increasing in density is not tested. Hence, differential responses to density could be an important first step in the identification of invasiveness or in determining traits associated with spread. Here, we propose a simple experimental method using the biogeographical filter as a first approximation to test whether there is evidence for differences in an invasive species in any response characters in competition when introduced to more than one novel region.

Competition in plants is a fundamental concept tested and used extensively as a tool to understand population dynamics, patterns of diversity, and community composition with literally over 6000 papers published within the last 10 years on the topic (Web of Science V.4.3, query ‘plant competition’). As such, it is reasonable to propose that at some level use of simple competition experiments can also potentially elucidate mechanisms associated with an introduced species becoming invasive. Certainly, the success of plant species when introduced is not necessarily attributable solely to changes in competitive effects or responses, but we propose that, regardless of the reason for the success of some invasives, simple competition experiments within a single invasive plant species from different regions (i.e. intra-specific but inter-regional) can test whether there is evidence for differences in competitiveness associated with density when introduced (provided seeds are collected widely from each region and sample more than one region). If the reason for success is not related to density when introduced, then there is no reason to expect that competition between individuals from the same region should differ from competition between individuals from different regions under controlled conditions. In summary, we predict that competition between individuals of invasive plant species from different novel regions is a useful first step in screening invasive plant species. We use a highly successfully invader, *Centaurea solstitalis* or yellow starthistle, to test the prediction that differentiation (either due to sampling effects or local adaptation) leads to relative differences in competition within and between novel regions using standard pot-based competition experiments in the greenhouse.

## Methods

### Study species

*Centaurea solstitalis* is a highly invasive weed from Eurasia [16]. It is a prolific seed producer with up to 1000 s of seeds produced per plant [17], 125–250 millions seeds per hectare reported in an invaded region [18], produces two types of seeds – pappus and non-pappus each possessing unique dormancy attributes [19], [20] - all making it a perfect candidate to explore the importance of density and competition. In California, it is reported in 56 of 58 counties [21], and in Argentina, it is also highly invasive and widespread [6].

### Experimental Design

Seeds of *C. solstitalis* were collected widely from seed heads in 2005 from 10 populations in each of the two introduced regions, California and Argentina [4], sorted into pappus and non-pappus, and thoroughly mixed within region by each seed type. General differences between regions in seed ecology and the importance of competitive effects intra and inter-regionally were compared using density series in a greenhouse at York University, Canada. These two levels of contrast were tested by sowing seeds in 15 cm diameter pots with standard potting mix in the following density series: 1, 2, 5, 10, and 20 seeds per pot using seeds from only one region and both regions mixed at a 50∶50 ratio (modified replacement series, i.e. proportion varied, 100% or 50%, with density held constant but more than one density tested, [22], [23]). Ten replicates per density per region per seed type were tested (pappus and non-pappus seeds were tested independently). A standardized grid-based planting was used to ensure identity of each seed and subsequent seedling, and initial application of water was done carefully to ensure that seeds did not move prior to germination. Germination, relative growth rate (rgr) of individuals (total number of leaves recorded weekly from emergence date for each individual and biomass at the end of the growing season by total number of days since emergence), and survival were recorded for the span of the experiment (4 months total, ended when the first individuals flowered in the greenhouse September 15^th^ 2007). Water (added to saturation every 2–3 days), nutrients (20∶20∶20 NPK added at onset), and light were not limiting in this experiment. All plants were harvested, dried for 48 h at 60°C, and weighed.

### Statistics

The replicated regression design used here (10 reps per density per region per seed type) permits two set of analyses to test for general differences between regions [23], [24]. Firstly, broad-scale patterns were identified using generalised linear models [25], and factors identified as significant (alpha set at p<0.01 to control for table-wide errors [26]) were further tested for density dependence via simple regressions of the mean summary data per level. Additional analysis is necessary since a direct relationship between density and a response variable does not necessarily imply that there is density dependence, i.e. more seeds should equal more plants. Only when there is a disproportionate (i.e. curvilinear) increase or decrease in the response with density do we infer density dependent regulation. Non-linearity for population level measures such as proportionate germination or survival thus indicates density dependence (with an increase of r^2^ of at least 10%), and for individual plant measures such as rgr, a slope significantly different from 0 indicates density effects [27], [28]. Secondly, to test for differences in the mean competitive effects of individuals [29] from the same region versus individuals mixed with different regions, the ‘relative interaction index’ or Rii was calculated for each of the response measures recorded [30]. This index is a direct measure of effect size and is calculated as following:
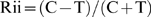



Controls are designated as performance in monocultures (i.e. seeds from same region) and treatments as the individuals grown in mixture with seeds from the second region at every density. The metric ranges from +1 to −1 with negative values indicating competition and positive indicates facilitation, and two-tailed t-tests are used to test for differences from 0 (i.e. no relative difference in the effect of neighbours in mix to mono, alpha also set at 0.01). The net differences in actual density between mixture and monocultures was also tested directly as a predictor of effect size (i.e. 10 seeds sown but 5 germinate in monoculture and 8 in mixture and the difference between the two treatments in actual density changes the competitive environment experienced by individuals therein).

## Results

### Density dependence and broad-scale patterns of seeds from the two invaded regions

In monocultures, seeds from California expressed significantly greater germination relative to Argentina under these controlled conditions (Table 1, CA: 65%+/−3%, AR: 41%+/−4%). Survival to final census also significantly differed between regions in favour of California (Table 1, CA: 70%+/−3%, AR: 60%+/−4%). There were no differences in any measure by seed type (pappus/non-pappus, all GLMs p>0.05) nor an effect of census on any measure, i.e. timing of germination did not differ (GLMs p>0.05).

**Table 1:** A summary of the generalised linear models used to test the importance of density, region, and density by region on the four responses measured in this greenhouse experiment of *C. solstitialis*.

Measure	Factor	DF	Chi-square	Prob>Chi-square
Germination	Density	3,78	6.55	**0.01**
	Region	1,78	16.77	**0.0001**
	Density×Region	3,78	12.77	**0.0004**
RGR leaves	Density	3,196	2.98	0.08
	Region	1,196	1.1	0.3
	Density×Region	3,196	4.11	0.04
RGR mass	Density	3,152	3.73	0.05
	Region	1,152	2.37	0.12
	Density×Region	3,152	5.55	0.018
Survival	Density	3,78	70.75	**0.0001**
	Region	1,78	4.97	**0.0001**
	Density×Region	3,78	4.49	0.03

Seeds collected from two invaded regions were tested (California and Argentina), densities included 1, 2, 5, 10, and 20 seeds per pot, and details for the responses are reported in the text. Proportionate germination and survival were tested with logistic models and relative growth rates (rgr) with linear models. Bold denotes significant effects.

**Figure 1:**
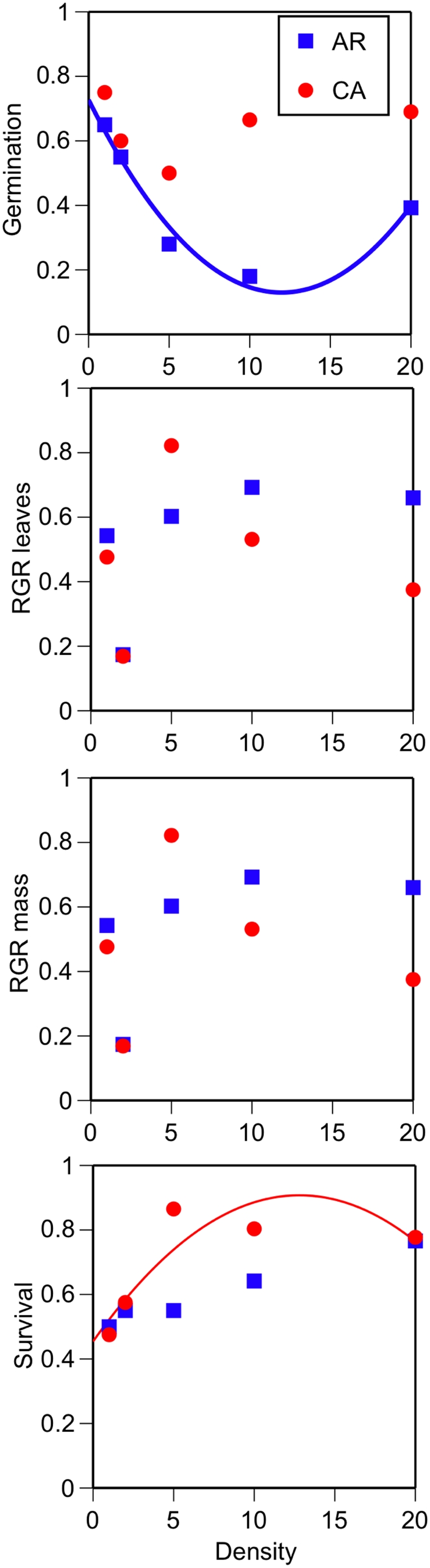
The importance of density on measures of the invasive weed *C. solstitialis*. See text for generalized linear model statistics. Data is summarized by plotting the mean response per seed densities tested (1, 2, 5, 10, & 20).

Germination and survival of *C. solstitialis* significantly responded to changing seed densities (Table 1). Negative density dependence was detected in the germination of seeds from Argentina (Table 1, significant density×region effect, Fig. 1, best fit curvilinear r^2^ = 0.97 on summarized data) while survival of plants responded positively at first to increasing seed densities but then began to decrease – particularly for the California populations (Fig. 1, best fit curvilinear r^2^ = 0.77 on summarized data).

#### Intra versus inter-regional differences in competition

**Figure 2:**
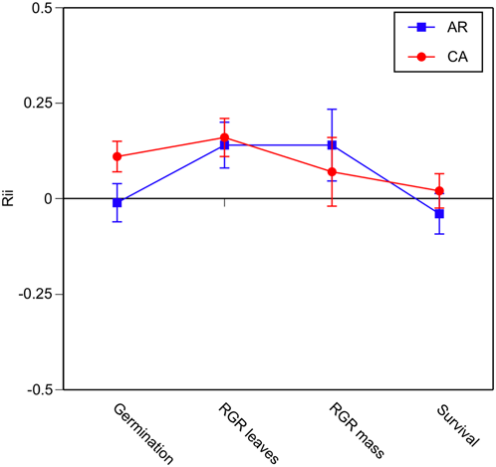
The relative effect of growth in competition with *C. solstitialis* plants from another invaded region (California and Argentina) to performance in monocultures, i.e. seeds from the same region. The mean relative interaction indices are plotted (Rii)+/−1 s.e. and were calculated for each paired density (2, 10, & 20 seeds per pot).

**Figure 3:**
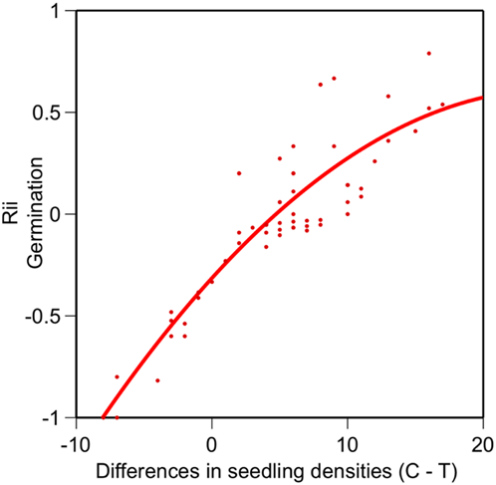
Regression of the relative differences in density of germinants of *C. solstitialis* from California when planted with only seeds from the same region or mixed with *C. solstitialis* seeds from Argentina and the relative interaction indices for germinants from this region. Control (C) refers to individuals grown in monoculture (California only) and the paired treatment (T) to the mixture of two regions. The fit curve is described by y = −0.03+0.06*x−0.004*(x−3.8)^2^ (r^2^ = 0.37 and p = 0.0001).

There was no evidence for differential competitive effects of *C. solstitialis* collected from Argentina when grown in mixtures with seeds from California (Table 2, Fig. 2). However, seeds from California had significantly greater germination when grown in monocultures than with seeds from Argentina, and also expressed a relatively higher rate of rgr leaves in the absence of intra-regional competition (Table 2, Fig. 2). In the former instance, the difference in the density of germinated seedlings between treatments positively predicted the strength of the relative interaction indices for the germinants from California (Fig. 3).

**Table 2:** Tests of the relative interaction index (Rii) contrasting performance in mixtures to that in monocultures.

Region	Measure	DF	t	p	sign
**AR**	Germination	96	−0.21	0.83	0
	RGR leaves	96	2.27	0.03	0
	RGR mass	57	1.45	0.15	0
	Survival	96	−0.7	0.5	0
**CA**	Germination	100	2.75	**0.007**	**+**
	RGR leaves	100	3.24	**0.001**	**+**
	RGR mass	58	0.8	0.42	0
	Survival	100	0.42	0.68	0

Mixtures refers to the performance of individuals of *C. solstitialis* in competition with seeds sown from two regions, Argentina (AR) and California (CA), at 2, 10, and 20 seeds per pot or in monocultures, i.e. seeds from only one invaded region. Two-tailed t-tests were used to determine whether the mean Rii values were significantly different from 0 (at p<0.01).

## Discussion

Hypotheses explaining the relative success of invasive species span the entire spectrum of population and community ecology including evolutionary arguments such as local adaptation, i.e. evolution of increased competitive ability, EICA [31], [32] to more stochastically driven processes such as disturbance [33], [34] or pure sampling effects such as propagule pressure [35]. Nonetheless, these hypotheses need not be mutually exclusive, but it would be useful to be able to sort invasive species with simple, standardized experimentation into at least the most broad set of potentially applicable hypotheses [2], i.e. is there evidence for differences in the competitiveness of the species or is opportunity/and or the local environment likely causal. Here, we successfully tested whether competition between individuals of an invasive species from different regions is sensitive to mixed versus monocultures by region.

*Centaurae solstitialis* from one the two invaded regions, Argentina, did not differ in performance in the intra versus inter-regional comparisons which cursorily suggests that competitive ability has not changed in this region. Hence, hypotheses related to stochastic processes such as disturbance might be more powerful explanatory avenues of research, and evidence to date indicates this is likely the case [6]. However, *C. solstitialis* from California performed relatively better in monocultures for some key responses which suggests that either local adaptation has occurred or founder effects sampled individuals, and now populations (10 sampled throughout region), with divergent competitive abilities (i.e. increased germination and leaf growth rates in California comes at a cost when in competition with plants from Argentina which continue to adopt a more conservative strategy). This is not to say that disturbance is necessarily unimportant in California [6], but that it is clear that individuals of *C. solstitialis* differ in this region in the expression of traits in the context of plant competition. Interestingly, greater differences in the actual densities of seedlings recorded in the paired monoculture versus mixture pots positively predicted the effect size estimates for germination in California. These relative increases suggest that germination in the field is likely not regulated at all by potential increases in the density of other *C. solstitialis* in California within the local neighbourhood (i.e. within a 15 cm range). Admittedly this is a simplistic first approximation to understanding the dynamics of invasive plant species and the myriad of causal factors, but it did clearly demonstrate that differentiation within an invasive species is detectable using competition experiments.

Conceptually, this experimental approach is highly novel as competition between invasive and native species has been tested [36]–[38] but not within the invasive species directly [39]. Importantly, even if no difference is detected, this experimental design (i.e. cage matching an invasive species) has biological relevance in that it points towards explanations that focus more on disturbance or environmental drivers such as climate matching [40] rather than explanations that necessarily invoke change such as EICA or enemy release [41]. The outcome of the test does not thus determine the usefulness of the experiment, and these approaches are of course the best types of studies – even if preliminary. Furthermore, it challenges a dogma which seems to be common in the general perception of invasives in that if an introduced species is numerically dominant, i.e. increases in density, it must also always be a good competitor and free from regulation [35]. If the relative abundances of the invasive in different regions or locally are documented, this design can also be used more finely to assess whether there is evidence for differential competitive effects as related to dominance or density (*sensu* Goldberg 1996).

Another strength of coupling standard plant population ecology experiments with biogeography is that the relative importance of density dependence can be inferred, seed biology described, and broad differences in the relative importance of life-stage screened. In this particular invasive species, the evidence concurs with field studies describing the importance of life-history traits and the seed biology of *C. solstitialis* in California in that a large proportion of seeds can germinate and frequently do so very quickly [18]. While not measured directly here, it is likely that *C. solstitialis* can express the phenomenon described as adaptive acceleration in competitive contexts [42]. In this study, increased germination by seeds from California was detected, and acceleration was potentially expressed via an increased relative growth rate similar to field studies [13]. Biogeographically, the differences in density dependence between Argentina, negative effects on germination, and California, higher germination and survival and even positive effects of initial increases in density, clearly suggest that in Argentina prolific seed production and subsequent seed and seedling densities do not benefit this species in an intra-specific competitive context here while in California it does not come at a cost. This strongly suggests that *C. solstitialis* in California is either able to capitalize on opportunity via high seed densities or is positively influenced by increasing local abundances of its seed. Few studies of density dependence fail to detect negative effects on germination [27], [28], and this is thus a unique finding suggestive of an important trait related to invasiveness. Hence, experimentally pairing an invasive plant species from different regions not only facilitated the detection of evidence for potential differentiation by region but provided a clear signal of the relative importance of seed and seedling densities within each invaded region.
